# Exploring the Potentials of Membrane Gas Separation for CO Concentration After Plasma Catalytic CO_2_ Splitting

**DOI:** 10.3390/membranes15120380

**Published:** 2025-12-13

**Authors:** Daria Miroshnichenko, Evgenia Grushevenko, Maxim Shalygin, Dmitry Matveev, Ilya Borisov, Anton Maximov, Stepan Bazhenov

**Affiliations:** 1 A.V. Topchiev Institute of Petrochemical Synthesis RAS, Leninsky Pr., 29, Moscow 119991, Russia; dmiroshnichenko@ips.ac.ru (D.M.); mshalygin@ips.ac.ru (M.S.); dmatveev@ips.ac.ru (D.M.); boril@ips.ac.ru (I.B.); sbazhenov@ips.ac.ru (S.B.); 2 Department of Chemistry, Lomonosov Moscow State University, Leninskie Gory, 1, Moscow 119991, Russia; max@ips.ac.ru

**Keywords:** carbon dioxide, carbon monoxide, membrane gas separation, polymeric membranes, plasma catalytic CO_2_ splitting to CO

## Abstract

Today, reducing carbon footprints requires the development of technologies to utilize CO_2_, particularly by converting it into valuable chemical products. One approach is plasma-catalytic CO_2_ splitting into CO and O_2_. The task of separating such a ternary mixture is nontrivial and requires the development of an efficient method. In this paper, we have developed a comprehensive scheme for the separation of a CO_2_/CO/O_2_ mixture using membrane technology. The novelty of this work lies in the development of a complete scheme for separating the products of plasma-chemical decomposition of CO_2_ to produce a CO concentrate. The calculations utilized the principle of a reasonable balance between the recovery rate and the energy consumption of the separation process. This scheme allows production of a CO stream with a purity of 99%. To achieve this goal, we have proposed the sequential use of CO_2_-selective membranes based on polysiloxane with oligoethyleneoxide side groups (M-PEG), followed by polysulfone (PSF) hollow-fiber membranes to separate CO and O_2_. For these membranes, we measured the CO permeability for the first time and obtained the selectivity for CO_2_/CO and O_2_/CO. The potential of membrane separation was demonstrated through a three-stage process, which includes recycling of the CO removal stream and concentration after CO_2_ plasmolysis. This process was calculated to yield a highly pure CO stream containing 99 mol% with a recovery rate of 47.9–69.4%. The specific energy consumption for the separation process was 30.31–0.83 kWh per 1 m^3^ of feed mixture, and the required membrane area was between 0.1 m^2^ for M-PEG and 42.5–107 m^2^ for PSF, respectively.

## 1. Introduction

CO_2_ capture processes can be divided into four main types: pre-combustion, post-combustion, oxy-fuel combustion, and chemical looping [1,2,3,4,5,6,7]. Post-combustion capture is the most common method [8]. The principle of this method has been used for more than 80 years in natural gas sweetening, industrial production of chemicals, etc. The following technologies are considered:

(1) Chemical absorption using amine solutions is a well-established and widely used CO_2_ capture technology that falls within the Technology Readiness Levels (TRL) 9 range, as defined by the classification of CCUS methods (CSLF Technology Roadmap 2021 [Electronic resource]. Available online: https://fossil.energy.gov/archives/cslf/sites/default/files/CSLF_Tech_Roadmap_2021_final_0.pdf (accessed on 21 June 2025). It has been widely used for decades and is currently used in a number of projects around the world for power generation and in industry [9,10,11,12].
(2) Physical separation is based on adsorption, absorption, cryogenic separation/dehydration, and compression. This capture method is also related to TRL9. Solid adsorbents such as activated carbon, aluminum oxide, metal oxides, and zeolites are used for physical adsorption [13]. CO_2_ desorption is realized by increasing the temperature (TSA), pressure (PSA), or using a vacuum (VSA) [14,15,16]. Organic solvents are used for physical absorption (a mixture of dimethyl esters of polyethylene glycols in the Selexol process and methanol in the Rectisol process, etc.) [17,18,19]. The CO_2_ desorption process is achieved through solvent decompression. Physical separation is primarily used in the natural gas processing and the ammonia industry.
(3) The membrane technology for CO_2_ capture is currently under R&Ds and falls within TRL6–TRL7 [20,21,22,23]. Membrane gas separation is based on the use of CO_2_-selective membranes with the following advantages: the absence of first-order phase transitions, environmental friendliness, modularity, compactness, ease of maintenance and upscaling, the ability to use electricity only, flexibility, and the possibility of integration with other separation processes. MTR has developed a series of CO_2_-selective Polaris membranes and conducted a number of laboratory, pilot-scale, and demo tests that have proven >90% capture efficiency without chemical reactions and VOC emissions (MTR Carbon Capture Technology, available online: https://mtrccs.com/technology/ (accessed on 26 June 2025)).

Despite the obvious successes in the development of CO_2_ capture technologies, these are not enough to fully implement the carbon capture, utilization, and storage (CCUS) strategy [24]. The next step is the development of technologies for the use of CO_2_ in high-value-added products [4,25,26]. CO_2_ can be applied as a raw material to produce fuels and their components [27,28,29,30,31,32], chemicals [33,34,35,36,37,38,39], polymers [40,41], and building materials [42]. This approach contributes to the development of a circular economy by reusing captured carbon and reducing dependence on fossil fuels.

However, to produce marketable products from CO_2_, it is often necessary to convert it into CO in a significant number of cases. The development of CO_2_ decomposition technologies has the potential to establish a platform for a carbon-neutral economic model in the future. CO is widely used in various industries, including the production of hydrocarbons, methanol, acetic acid, metal carbonyls, phosgene, etc. [43,44,45,46].

The main challenge in CO_2_ splitting is to overcome the thermodynamic stability of the CO_2_ molecule and activate it. A significant amount of energy is needed to break the O=C=O double bonds and dissociate the molecule [47]. To meet this challenge, different approaches and technologies are being developed: catalytic, plasma–chemical [48,49,50], photochemical [51,52,53], biochemical [54], thermochemical [55], and electrochemical [56]. Plasma–chemical splitting of CO_2_ is a promising technology, as it is induced by a plasma discharge and can occur at low temperatures. Moreover, plasma–chemical CO_2_ splitting can be carried out using alternative energy sources, which increases the economic efficiency of the process. This eliminates the possibility of a reverse reaction (1) [48,49,50]:

(1) $$2{\mathrm{CO}}_{2}↔2\mathrm{CO}+O_{2}$$

(ΔH_298K_ = +282.8 kJ/mol; ΔG_298K_ =
+257.2 kJ/mol)

In the process of plasma-based CO_2_ conversion, gas separation is essential to facilitate CO utilization in subsequent processes. Separating these three gases is a challenge specific to plasma-based conversion technologies. By contrast, in solid-oxide electrolysis cells, only two gases (CO and CO_2_) need to be separated, which is much less challenging [57]. Absorption, cryogenic, adsorption, and membrane technologies for CO_2_ removal are currently at a high level of readiness [2,7,22,58]. The CO/O_2_ separation task is not trivial and requires careful consideration. These gas molecules have similar diffusion diameters, force constants, and boiling points [43,59].

There are two main industry technologies for CO separation (e.g., separation of CO/H_2_ mixture): cryogenic distillation and chemical absorption by Cu (I) salts [43,60]. However, if the gas mixture contains CO_2_ and O_2_, additional separation stages are needed. In this case, the use of cryogenic distillation is very energy-intensive due to the close boiling points of O_2_ and CO.

For this task, a concept for the stepwise adsorption separation of a CO_2_, CO, and O_2_ mixture has been proposed, and the fundamental feasibility of obtaining nearly pure component streams has been demonstrated through modeling [61,62,63]. However, an estimate of the energy costs was not presented. Pérez-Carbajo et al. [61] evaluated a range of zeolite materials using molecular simulations combined with ideal adsorption solution theory. Their study proposes a dual-pressure swing adsorption (PSA) configuration employing two different zeolites. In the first PSA unit, CO_2_ is separated using a FAU-type zeolite, while the second PSA unit utilizes a BRE-type zeolite to capture CO. Both PSA cycles operate at an adsorption pressure of 2 bar and a regeneration pressure of 0.1 bar, implemented within a four-bed process design. This configuration theoretically yields a CO product stream with a purity of 98.73%. However, it should be emphasized that these promising simulation-based results have not yet been validated experimentally.

Gas diffusion electrodes (GDEs) offer a theoretically viable approach for effectively separating oxygen from the product gas. A proof-of-concept demonstration has already been provided by Kaufmann et al. [64]; however, further development is required to raise the technology readiness level for industrial-scale applications. GDEs—commonly employed in the electrolyzer and fuel cell industries—are multilayer porous electrodes coated with a catalyst. Oxygen molecules permeate through the GDE, where they undergo catalytic conversion to hydroxide ions (OH^−^) and are subsequently transported toward the anode. Ultimately, a recombination reaction takes place, enabling oxygen removal from the system. A new approach to O_2_ extraction in a plasma-chemical CO_2_ decomposition reactor has been proposed, based on the use of perovskite membranes [65]. This method has a potential advantage for conducting the process at high temperatures, due to the special oxygen transport through perovskites. This allows for increased CO_2_ conversion, while the process is not effective at low temperatures [66].

In this study, we propose to use membrane gas separation instead of adsorption, as it is a compact, reagent-free, non-periodic process [67]. In terms of energy efficiency, these processes differ: for membrane gas separation, most of the energy is used for compressing or evacuating the gas streams. Adsorption, on the other hand, requires cycling between high and low pressure, as well as having a bypass stream for desorption. Previously, no general scheme for separating plasma-chemical decomposition products using membrane gas separation alone had been presented. Furthermore, the energy consumption and efficiency of the separation process had not been assessed.

In this context, it is relevant to explore the possibility of utilizing membrane technology in order to separate the mixture of products resulting from plasma-chemical CO_2_ splitting. In this study, we selected membranes and, for the first time, proposed and modeled a separation process flowsheet that produces pure CO as the final product.

## 2. Membrane Selection for CO_2_ Plasma Splitting Product Mixture Separation

Mass transfer through a gas separation membrane is typically described by the “solution–diffusion” mechanism. Based on this model, membranes can be designed with a predominant diffusion or sorption component for the transport of gases. Diffusion-selective membranes are usually made from low-permeable glassy polymers, while sorption-selective membranes are made from elastic or highly permeable glassy materials.

During the plasma-chemical CO_2_ splitting, incomplete conversion leads to the formation of CO, O_2_, and an unreacted CO_2_ mixture. CO_2_ has a small diffusion molecular diameter (0.302 nm) and is highly soluble in most polymer materials, resulting in preferential transport to permeate. Considering the need to recycle unreacted CO_2_ back into the reaction zone, a two-stage separation process may be more effective. In the first step, a concentrated stream of CO_2_ is separated from the mixture with high selectivity. In the second step, carbon monoxide is removed from the remaining gas.

CO_2_ separation with membranes is a topic that has been actively developing in recent years. A lot of studies were devoted to the development of both new membrane materials and engineering approaches to the CO_2_ capture from different gas streams [58,68,69]. Highly permeable partial-ladder polymers, including those with triptycene fragments in the main chain [70], thermally rearranged polymers [71], and и perfluorinated polymers [72] were demonstrated to have record CO_2_ permeability and ideal selectivity. However, these polymers exhibited plasticization at high CO_2_ concentrations and a reduction in transport properties over time due to the aging effect. Of particular interest are Pebax-based polymers [73], polymeric ionic liquids [74], and polymers with polyether fragments [75,76,77]. The increased solubility of CO_2_ in these polymers is explained by the specific interaction between quadrupole CO_2_ molecules and charged or dipole fragments in the polymer chains. However, the permeability of these materials is often low. In contrast, polysiloxane-based membranes exhibit high gas permeability [78,79,80], while introducing polyethylene oxide groups into the side chains allows for achieving adequate selectivity while maintaining high gas permeability [75,81,82]. In this regard, in this work, a membrane based on polysiloxane with side oligoester groups, M-PEG, was selected. The CO_2_ permeability coefficient (1300 Barrer) is similar to that of highly permeable polydimethylsiloxane (PDMS) at the selectivity CO_2_/N_2_ of 37 [82]. The transport properties of these polymers, as well as the fact that they belong to the class of siloxane rubbers, which are widely used in commercial membranes due to their stable transport properties, make them promising for the development of composite CO_2_-selective membranes. These membranes could then be used in the development of modules for carbon capture. Therefore, this material was considered for the first step of the CO_2_/CO/O_2_ separation scheme.

Checchetto et al. address the issue of membrane selection for CO_2_/CO separation [83]. It has been observed that the separation of CO_2_ and CO is determined by the differences in their sorption interactions with a polymer. Therefore, polymers with specific interactions with CO_2_ or with a facilitated transport of CO_2_ would be advantageous to the CO_2_/CO separation task.

However, the CO/O_2_ separation problem has not previously been studied in detail, with the exception of work [84]. A comparative table of the permeability coefficients of CO_2_, CO, and O_2_, which are the target gases in this study, as well as the corresponding selectivities, is presented below (Table 1).

Polysulfones (PSF) are amorphous, glassy polymers featuring an aryl–aryl linkage that imparts exceptional toughness and thermal stability. These polymers exhibit excellent chemical inertness and are resistant to hydrolysis, heat, and oxidation. Polysulfones are commonly employed as membrane materials in various separation processes, including water purification, biofuel recovery via pervaporation, and gas separation [87]. PSF is a well-known membrane material. Different scientific groups study its gas separation properties. The CO_2_ permeability coefficient of a polymer film depends on its chemical structure and pretreatment. It varies between 4.6 and 6.4 Barrer [87,88,89,90,91,92]. McCandless investigated gas transport through a Sulfone 47 film (produced by Union Carbide) and found CO permeability coefficients: 0.37 Barrer at 30 °C and 1.2 Barrer at 100 °C [93]. PRIZM air separation membranes (air products) made of polysulfone have been proposed to adjust the hydrogen-to-CO ratio in syngas production [43].

Based on M-PEG8 and PSF materials, gas separation membranes were developed, and the CO_2_/CO/O_2_ permeability of these membranes was studied for the first time. In this work, we propose a comprehensive scheme for the first time to obtain CO (99% purity) from the products of plasma-chemical decomposition of CO_2_ based on membrane gas separation, including the recovery of unreacted CO_2_. The novelty of this work lies in the development of a complete scheme for separating the products of plasma-chemical decomposition of CO_2_ to produce a CO concentrate. The calculations utilized the principle of a reasonable balance between the recovery rate and the energy consumption of the separation process.

## 3. Materials and Methods

### 3.1. Membranes

#### 3.1.1. CO_2_-Selective Membrane Based on Oligoethylene Glycol Methyl Ether Substituted Polysiloxane (M-PEG)

Polyorganosiloxanes with oligoethylene glycol–containing side groups (PEG8) were synthesized through a polymer-analogous transformation of polymethylhydrosiloxane (PMHS) using a hydrosilylation reaction, following the one-step approach described in [82]. PMHS (ABCR, Karlsruhe, Germany, Mn = 1900 g/mol) was dissolved in toluene (chemical grade, Chimmed, Podolsk, Russia), then methyl heptaethylene glycol allyl ether was added, and in the presence of Pt-based Karsted catalyst (ABCR, Karlsruhe, Germany) solution was stirred for 2 h at a temperature of 60 °C. After that, allyl-terminated PDMS (ABCR, Karlsruhe, Germany, Mn = 28,000 g/mol) at a concentration of 0.2% to substituted polysiloxane was added to the reaction mixture. The resulting mixture was stirred for 1 h. After this, PMHS was added at an equivalent concentration to PDMS, and the reaction continued for an additional hour. The polymer membrane was obtained by pouring a 15% wt. spinning solution in toluene onto polyacrylonitrile porous support. The thickness of the resulting membrane was 1.5 ± 0.5 μm.

#### 3.1.2. Hollow-Fiber Polysulfone Membrane (PSF)

For hollow-fiber membrane formation, the spinning solution of polysulfone (ULTRASON^®^ S 6010, BASF, Ludwigshafen, Germany) was prepared. N-methyl-2-pyrrolidone (NMP, Acros Organics, Geel, Belgium) was used as a solvent. The polymer concentration in the spinning solution was 24% wt. The solution was stirred at room temperature for 16 h to completely homogenize them. After that, it was filtered through a stainless-steel mesh under a nitrogen pressure of 1.8–2.0 bar.

PSF hollow-fiber membranes were obtained using a dry-wet phase inversion technique in a “free-spinning” mode. In this process, a spinneret with an outer and inner diameter of 0.8 and 0.5 mm, respectively, was used. Distilled water was used as an inner non-solvent (bore liquid). The spinning parameters were chosen as follows: a pressure above the spinning solution of 200 kPa, a flow rate of the bore liquid of 0.45 mL/min, and an air gap of 1 m. After spinning, the membranes were placed in water for at least five days. They were then dried in air at room temperature and 60% relative humidity for 24 h. The membranes were conditioned at 100 °C for 3 h before forming the membrane module to stabilize the transport properties.

#### 3.1.3. Membrane Morphology Study

The membrane structure and morphology were studied using scanning electron microscopy (SEM) micrographs. Micrographs were acquired using Thermo Fisher Phenom XL G2 Desktop SEM (Thermo Fisher, Waltham, MA, USA). Membrane cleavages were obtained after pre-impregnation of the membranes in isopropanol and subsequent fracture in liquid nitrogen. Using a Cressington 108 auto sputter coater (Cressington, Watford, UK), a thin (5–10 nm) silver layer was deposited on the prepared samples in a vacuum chamber (~0.01 mbar). The accelerating voltage during micrograph acquisition was 15 keV. The average thickness of the selective layer was determined from the obtained micrographs using Gwyddion software (ver. 2.53).

### 3.2. Gas Transport Properties of Membranes

**Figure 1:**
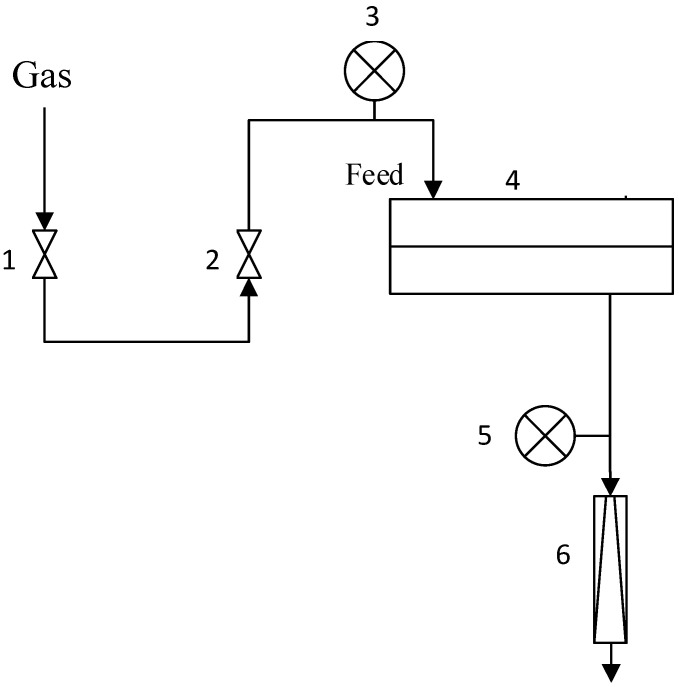
Lab set-up of gas permeance measurements: 1—feed on/off valve, 2—pressure regulator, 3—feed pressure gauge, 4—stainless steel module, 5—permeate pressure gauge, 6—permeate bubble mass flowmeter.

Gas permeation (CO, CO_2_, and O_2_) of the membranes was determined by the variable volume/constant pressure method. All experiments were conducted at room temperature (23.6–25 °C) and upstream pressure 0.5–30 atm. Downstream membrane pressure was atmospheric (near 1 atm). The flat sheet membrane was placed in a stainless-steel module with an active membrane area of 18.1 cm^2^. Permeating flux was measured by a bubble mass flowmeter in the lab setup (Figure 1). The permeance of the composite membrane was determined from the first derivative of the dependence of the permeating flow on the pressure drop. The standard deviation of experimental data was below 5%.

The permeance was calculated according to the formula:

(2) $$\frac{P}{l}=\frac{Q}{p\cdot S}$$

where (*P*/*l*)*_i_* is the permeance of the *i*-th individual gas, m^3^/(m^2^·h·atm); *Q* is the volumetric flow rate of the gas that passed through the membrane, m^3^/h; *p* is the transmembrane pressure, atm; and *S* is the membrane surface area, m^2^.

The ideal selectivity α for the *X/Y* gas pair was calculated using the equation:

(3) $$\alpha =\frac{{\left(P/l\right)}_{X}}{{\left(P/l\right)}_{Y}}$$

### 3.3. Mathematical Modeling of Ternary Mixture Separation

During plasma catalytic CO_2_ splitting into CO, a ternary gas mixture was obtained. Based on the data from references [94], where the conversion rate was not higher than 20% and no coke was formed, it is possible to calculate the composition of the gas mixture as follows: 18 mol% CO, 9 mol% O_2_, and 73 mol% CO_2_. We carried out mathematical modeling of a membrane separation scheme with compression of the initial gas flow to 1–30 bar. In this study, the flow arrangement was performed in countercurrent mode, as this ensures the most efficient separation [94]. Initial data for mathematical modeling are presented in Table 2.

**Figure 2:**
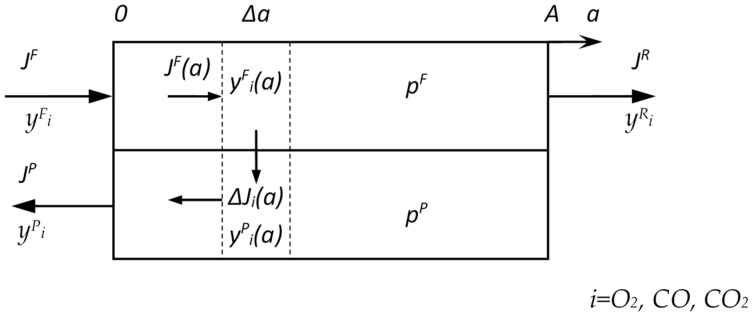
Scheme of counter-current mode of mass transfer calculation in the membrane module used in the modeling.

The mathematical model includes the following assumptions: isothermal conditions; plug flow in the feed membrane module channel; plug flow in the permeate membrane module channel for counter-current mode; and constant membrane permeance throughout the membrane module (Figure 2).

The system of equations was solved numerically using the finite difference method.

(4) $$\left\{\begin{array}{l} \Delta J_{i}^{F}(a)=-Q_{i}\left(p^{F}y_{i}^{F}(a)-p^{P}y_{i}^{P}(a)\right)\Delta a\\ \Delta J_{i}^{P}(a)=-Q_{i}\left(p^{F}y_{i}^{F}(a)-p^{P}y_{i}^{P}(a)\right)\Delta a\\ y_{i}^{F}(a)=\frac{J_{i}^{F}(a)}{\sum J_{i}^{F}(a)}\\ y_{i}^{P}(a)=\frac{J_{i}^{P}(a)}{\sum J_{i}^{P}(a)}\\ J_{i}^{F}(0)=J^{F}y_{i}^{0}\\ J_{i}^{P}(A)=0 \end{array}\right.$$

Stage cut and recovery rate for CO_2_, CO, and O_2_ were calculated using the following Equations (5)–(8):

(5) $$\theta =\frac{J^{P}}{J^{F}}$$

(6) $$\theta {\mathrm{CO}}_{2}\;\;\frac{J^{P}{\;y}_{\mathrm{CO}2}^{P}}{J^{F}\;y_{\mathrm{CO}2}^{F}}$$

(7) $$\theta \mathrm{CO}\;=\;\frac{J^{P}{\;y}_{\mathrm{CO}}^{P}}{J^{F}\;y_{\mathrm{CO}}^{F}}$$

(8) $$\theta O_{2}\;=\;\frac{J^{P}{\;y}_{O2}^{P}}{J^{F}\;y_{O2}^{F}}$$

### 3.4. Specific Energy Consumption Calculation

The calculation of the specific compression work of the polytropic process of the compressor was carried out using the Formula (9) [95]:

(9) $$W=\frac{n}{n-1}RT_{1}[({\frac{p2}{p1})}^{\frac{n-1}{n}}-1],\;[\frac{kJ}{kmol}],$$

where *n*—polytropic index (*n* = 1, 4); *T*_1_—inlet flow temperature, K; *p*_1_, *p*_2_—inlet and outlet pressures, respectively, atm; and *R*—universal gas constant, kJ/(kmol K).

Taking into account the compressor efficiency of 0.8 and heat losses in the engine of 0.1, the power consumed is calculated as [95]:

(10) $$W=\frac{Wshaft}{\eta_{engine}\cdot \eta meh},\;[\mathrm{kW}\cdot h],$$

where *W_shaft_*—motor power on the shaft, kW h; *η_engine_*—motor efficiency, and *η_mech_*—mechanical efficiency of the compressor.

An estimated calculation of specific energy (*Espec*) was carried out, considering the required power for a vacuum pump and compressor as the main consumers. The calculated values were compared with the initial flow of the gas mixture using the formula:

(11) $$Espec=\;\frac{W\cdot J^{C}}{J^{F}},\;[\mathrm{kWh}/m^{3}\;(\mathrm{initial}\;\mathrm{flow})],$$

where *J^C^*—compressible flow, kmol/s; and *J^F^*—initial flow, m^3^/h.

Calculated values can be used as the basis for a preliminary economic assessment for the estimation of the OPEX and CAPEX for the considered process.

## 4. Results and Discussion

### 4.1. Membrane Permeance

**Figure 3:**
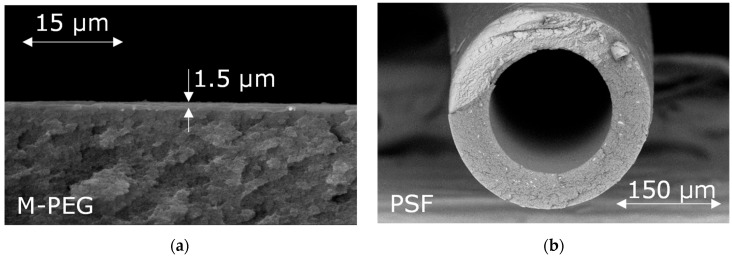

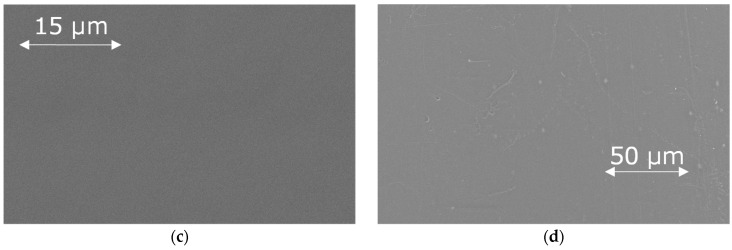
Cross-section and upper surface of M-PEG membrane (**a**,**c**) and hollow-fiber PSF membrane (**b**,**d**).

For CO_2_ separation, M-PEG-based composite membranes on a porous ultrafiltration support (PAN, HZG) with a selective layer thickness of 1.5 μm were obtained (Figure 3a). For CO concentration, a polysulfone (PSF) based hollow fiber gas separation membranes with an outer fiber diameter of 370 ± 5 μm and a wall thickness of 60 ± 5 μm were obtained. An SEM image of a cross-section of the hollow-fiber membrane is shown in Figure 3b.

The gas permeability of the obtained membranes for individual gases CO_2_, CO, and O_2_ is presented in Table 3. It is worth noting that the obtained membrane permeability values are reproduced in a series of experiments, including after operating the membranes at pressures from 1 to 30 atm. The gases being analyzed do not irreversibly interact with polysulfone or polysiloxane. As expected, no changes in the membrane microstructure were detected in the micrographs after membrane pressure testing.

M-PEG membrane demonstrates high ideal selectivity: 14.5 and 29.1 for CO_2_/O_2_ and CO_2_/CO, respectively. Thus, CO_2_ will preferentially be transported through the membrane. The CO:O_2_ ratio in the retentate is expected to vary depending on the initial mixture that is being separated. The CO_2_ permeability of the obtained M-PEG membrane is comparable to the permeability of the commercial Polaris membrane from MTR, which is 1000 GPU [96]. The CO_2_/CO selectivity of the obtained M-PEG membrane (29.1) is not inferior to membranes based on PEBAX (8–30) [86,97,98,99]. It is worth noting that the M-PEG permeability coefficient for CO_2_ is 1300 Barrer, while the PEBAX permeability coefficients are 8–350 Barrer [86,97,98,99]. In this regard, the M-PEG membrane seems promising for CO_2_ recovery from the CO_2_ plasmolysis product mixture and was adopted as a basis for further modeling of the separation process.

The obtained hollow-fiber membrane PSF has CO_2_/N_2_ and O_2_/N_2_ selectivities that are adequate for an ideal polysulfone. The data obtained are consistent with those reported in the literature [100,101]. The PSF membrane permeance is similar to the permeance of PSF membranes presented in the work of Ng et al. (14 GPU) [102]. Permeability values were obtained for a stabilized membrane, which was conditioned at elevated temperatures. This method allowed us to obtain permeability values relevant to long-term operation of the membrane module, over 2000 h in duration [101,103].

### 4.2. Development of Membrane Gas Separation Scheme

Separating a ternary mixture of 18 mol% CO, 9 mol% O, and 73 mol% CO_2_ to produce CO concentrate is a complex task. Among the components of the mixture, CO_2_ has the highest permeability through membranes (Table 4). Since the CO_2_ content in the mixture is high, it can be concentrated to high purity in the permeate and then returned to the reactor. In this case, the calculation criterion is the concentration of CO_2_ in the flow that is returned to the reactor. Therefore, in the first stage of process modeling, a CO_2_-selective M-PEG membrane is considered. And in subsequent stages, CO concentrate is obtained. Each stage of the unified separation process is discussed below. The calculation criteria are justified based on the goals of the work.

#### 4.2.1. Stage 1: CO_2_ Concentration

**Figure 4:**
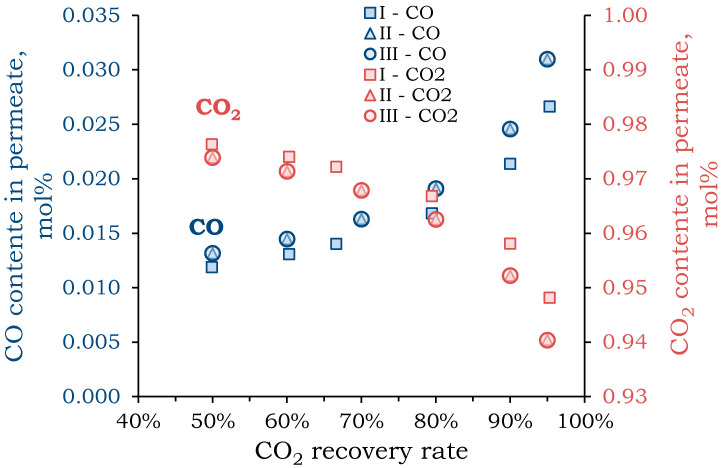
Dependence of the content of CO in the permeate (blue) and CO_2_ in the retentate (red) on the CO_2_ recovery rate at the first stage for different pressure mode: **I**—pressure above the membrane is 30 atm (square), pressure below the membrane is 1 atm; **II**—pressure above the membrane is 10 atm, pressure below the membrane is 1 atm (triangle); **III**—pressure above the membrane is 1 atm, pressure below the membrane is 0.1 atm (circle).

Based on the obtained data (Table 4), numerical modeling was performed using Equations (3)–(7) to calculate permeate and retentate compositions at varying CO_2_ recovery rates. An additional criterion for modeling the CO_2_ concentration stage was minimizing CO_2_ loss while maximizing CO_2_ recovery. Since CO_2_ plasmolysis is performed at atmospheric pressure, compressing the feed flow is necessary to create the driving force in membrane gas separation. In order to determine the optimal operating mode of the first stage membrane module, mathematical modeling of the separation process was carried out in three modes: **I**—pressure above the membrane is 30 atm, pressure below the membrane is 1 atm; **II**—pressure above the membrane is 10 atm, pressure below the membrane is 1 atm; **III**—pressure above the membrane is 1 atm, pressure below the membrane is 0.1 atm. The dependence of the CO concentration on permeate and the CO_2_ concentration in the retentate on the CO_2_ recovery rate was plotted to determine the optimal CO_2_ recovery rate (Figure 4). CO_2_ recovery rates below 50% were excluded, as the primary goal of the first separation step was to maximize CO_2_ recovery and return it to the plasmolysis reactor with minimal loss of the desired product.

As CO_2_ recovery increases, the concentration of CO in permeate also increases. The concentration of components in the flows has close values for all considered pressure modes. A significant increase in permeate CO concentration (by ~3%) was observed when CO_2_ recovery exceeded 90%. Similar results were obtained for oxygen. When CO_2_ recovery reached 90%, the oxygen concentration in permeate was ~2.3 mol%. Conversely, the CO_2_ concentration in the retentate decreased with increasing recovery, but a change in slope and a significant decrease in concentration were also observed when recovery exceeded 90%. This value, which allowed for the extraction of almost all CO_2_ (90%) with minimal loss of CO (~2.5 mol%), was chosen for further modeling.

**Figure 5:**
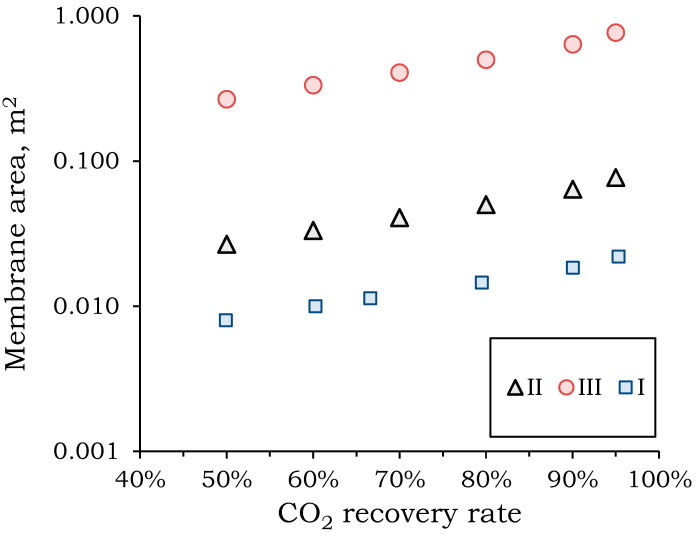
Dependence of the required membrane area on the CO_2_ recovery rate at the first stage of separation.

The recovery rate and pressure mode directly affect the required membrane area. The relationship between the required area and CO_2_ recovery for different pressure modes is illustrated in Figure 5.

**Figure 6:**
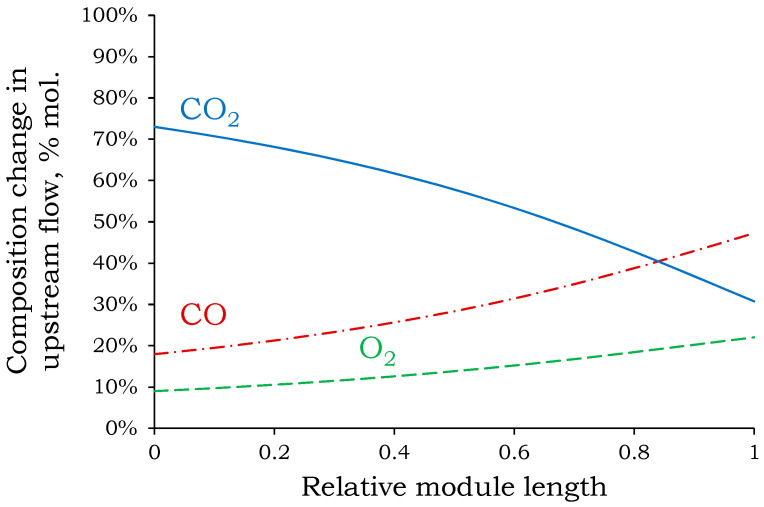
Change in concentrations of components above the membrane along the module length from inlet to outlet at the first stage.

Increasing the pressure drop across the membrane reduces the required membrane area by an order of magnitude. Reducing the membrane area will significantly reduce capital and operating costs for the separation process. The largest membrane areas are required for the vacuum system (mode **III**): 0.27–0.77 m^3^ to process a feed mixture flow of 1 m^3^/h at varying degrees of CO_2_ extraction. The smallest membrane area is required for a membrane pressure of 30 atm (mode **I**): 0.008–0.022 m^3^ to process a feed mixture flow of 1 m^3^/h at varying degrees of CO_2_ extraction. The higher the component is to be extracted, the larger the required area. In the recovery range from 90 to 100%, a sharp increase in the required membrane area of 57% is observed. This increase in area is comparable to the increase in area in the recovery range from 50 to 90%. This behavior is associated with a change in the concentration profile along the module length (Figure 6).

As shown in Figure 6, CO_2_ is the most permeable component, and its concentration above the membrane decreases sharply from the inlet to the outlet along the module length. Analysis of its concentration profile reveals that most of the CO_2_ was transferred to permeate in the first half of the module. Towards the end of the module, its concentration above the membrane continues to decrease sharply, resulting in a decrease in the difference in partial pressures and consequently a decrease in the driving force for the mass transfer process. As a result, more membrane area is required to approach a complete CO_2_ removal rate (approximately 100%). This explains the sharp increase in required membrane area for CO_2_ removal rates greater than 90%. The relative change in the required membrane area at 85% and 90% extraction rates is 11%. At 90% and 95%, the relative increase is 18%. With an additional 2% increase in extraction rate, the relative change in area increases to 35%. This confirms the appropriateness of the selected CO_2_ removal rate (90%) for the first stage.

To select the optimal pressure regime, it is necessary to consider not only the required membrane area but also the specific energy consumption. In the case of excess pressure above the membrane, energy is consumed to compress the flow. In the case of a vacuum system, energy is consumed to operate the vacuum pump for the permeate flow. A comparison of specific energy consumption and membrane areas under different conditions is presented in Table 4.

For simplicity, this article uses a feed flow rate of 1 m^3^/h for calculations. Scaling this flow rate will increase both membrane area and energy consumption. Although membrane cartridges require periodic replacement (usually once a year), the main contributor to operating costs is energy consumption. Therefore, it is important not only to operate with reasonable membrane areas but also to reduce energy consumption. Therefore, it appears optimal to conduct the first stage of separation in pressure mode **II** (10–1 bar), where specific energy consumption is 0.1 kW h per 1 m^3^ of feed flow, and the required M-PEG membrane area is 0.06 m^2^ for a feed flow rate of 1 m^3^/h.

In this case, the calculation was based on a pressure of 10 atm above the membrane and 1 atm below it. The calculation criterion at this stage was to achieve a CO_2_ recovery rate of 90%. The first stage permeate stream is 0.69 m^3^/h, which is enriched in CO_2_ at 95.8 mol%, and is proposed to be returned to the CO_2_ plasmolysis reactor. CO and O_2_ concentrations in permeate are 2.3 and 2.5 mol%, respectively. This first-stage retentate is still a ternary gas mixture (0.31 m^3^/h), with a residual CO_2_ concentration of 23.5 mol% and the key components for further separation—CO at 52.6 mol% and O_2_ at 23.9 mol%. Therefore, it requires the second step of the separation process.

#### 4.2.2. Stage 2: CO Concentration

In the second stage, the main task was to separate CO and O_2_. As CO is used as a raw material to produce many chemical compounds, it is important to obtain a stream with a high CO concentration (>99%) for further utilization. For this purpose, it was proposed to use a PSF hollow-fiber gas separation membrane, which has been developed in this study.

**Figure 7:**
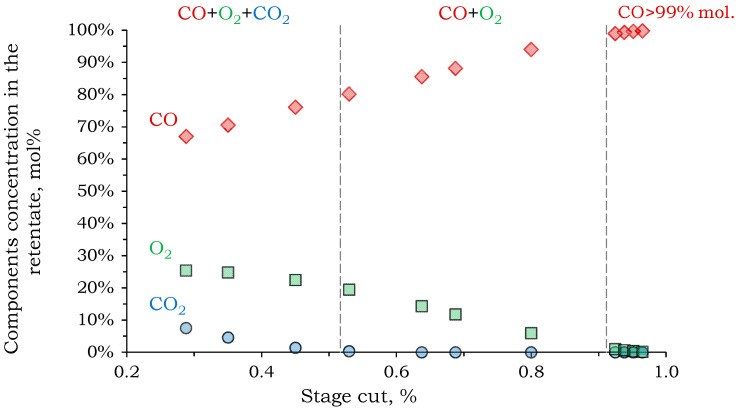
The dependence of the component concentration in the retentate on the stage cut of the second separation stage.

When modeling the second stage, we assumed that there was no pressure loss of the feed flow along the length of the module in the first stage (10 bar). The calculation criterion was to achieve a purity of 99 mol% for the target component (CO) in the retentate. The concentrations of CO, O_2_, and CO_2_ as a function of the recovery rate are shown in Figure 7.

Complete CO_2_ removal from the mixture in the second stage was possible with a recovery rate of more than 53%. As the recovery rate increased further, a binary gas mixture of CO and O_2_ remained in the retentate. As shown in Figure 7, in order to meet the specified calculation criteria (CO concentration in the retentate > 99%), a recovery rate of 93% is required. Therefore, the feed and permeate concentrations do not differ significantly.

According to calculations, 6.6 m^2^ of PSF membrane is required to achieve 99 mol% CO in the retentate stream in the second stage (0.02 m^3^/h). The permeate flow (0.29 m^3^/h) consists of 25.5 mol% CO_2_, 49.3 mol% CO, and 25.2 mol% O_2_. The CO removal rate from the stream is low (11%), and an additional separation stage is required to increase CO recovery.

#### 4.2.3. Stage 3: Increase CO Recovery Rate

**Figure 8:**
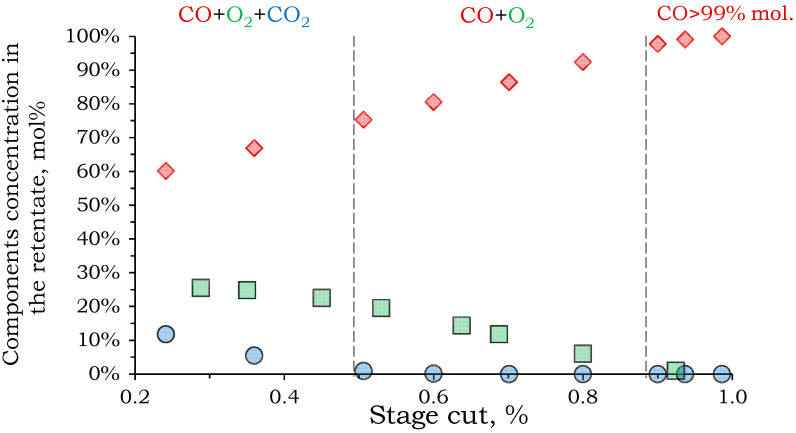
The dependence of the component concentration in the retentate on the stage cut of the third separation stage.

To increase the CO recovery rate, it was proposed to route the Permeate 2 stream to a module with PSF membranes for separation. Accordingly, the Permeate 2 stream, after preliminary compression to 10 atm, serves as the feed stream for stage 3. In the calculation, the same criterion was set as for stage 2: achieving CO purity in the retentate of 99 mol%. Figure 8 shows that achieving this target is only possible with a recovery rate above 93%.

The resulting flow distribution in the membrane module demonstrates that stage 3 membrane separation is close to stage 2: retentate flow–0.017 m^3^/h with 99 mol% CO, 1 mol% O_2_; permeate flow–0.272 m^3^/h with 26.8 mol% CO_2_, 46.2 mol% CO, 27.0 mol% O_2_ However, a large flow containing 0.126 m^3^/h of CO is released into the permeate, which is 69.8% of its initial amount.

Due to the high removal rate in the third stage, a permeate 3 stream containing CO_2_, CO, and O_2_ will be generated again, requiring an infinite repetition of similar stages to separate high-concentration CO. Therefore, it was decided to recycle permeate from the third stage back to the inlet of the second membrane module.

**Figure 9:**
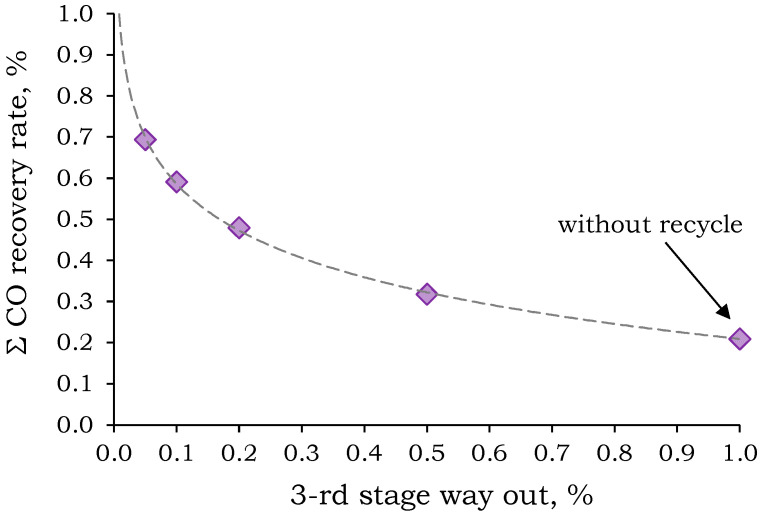
Dependence of the total CO removal rate on the permeate third stage way out stream.

To prevent the accumulation of CO_2_ and O_2_ in the system, it was suggested to discharge a portion of Permeate 3 (stage way out). However, a preliminary calculation was needed to determine the optimal stage way out stream in order to achieve a high CO recovery rate and minimize energy consumption and membrane area for the second and third separation stages. The dependence of the overall CO recovery rate on the stage way out stream is shown in Figure 9.

The resulting dependence is not monotonic and shows a tendency for the CO removal rate to increase sharply with decreasing blow-off stream. Figure 9 shows that in order to achieve a total CO recovery rate of ~50%, the blow-off stream should not exceed 20% of the permeate flux. With a 20% blow-off stream, the recovery is 47.9%, and the required PSF membrane areas for the second and third stages are 22.0 and 20.3 m^2^, respectively. Further increases in CO recovery rate up to 59.0% and 69.4% require a decrease in stage way out stream down to 10% and 5%, respectively. This leads to an increase in the required membrane areas in the second and third stages up to 35.2 and 33.3 m^2^ (for 10%), and 54.7 and 52.4 m^2^ (for 5%), respectively.

**Figure 10:**
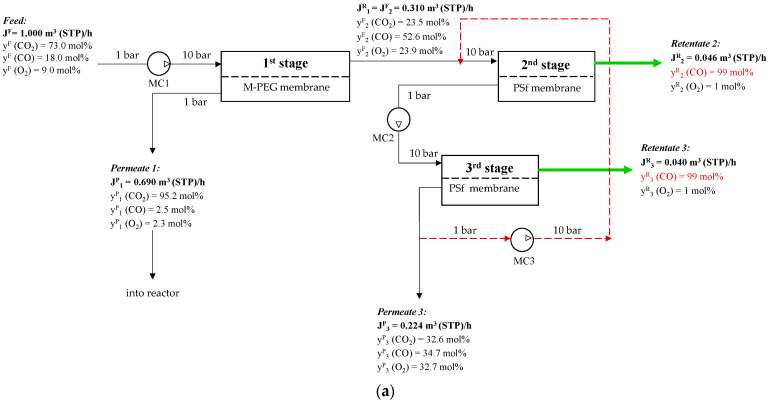

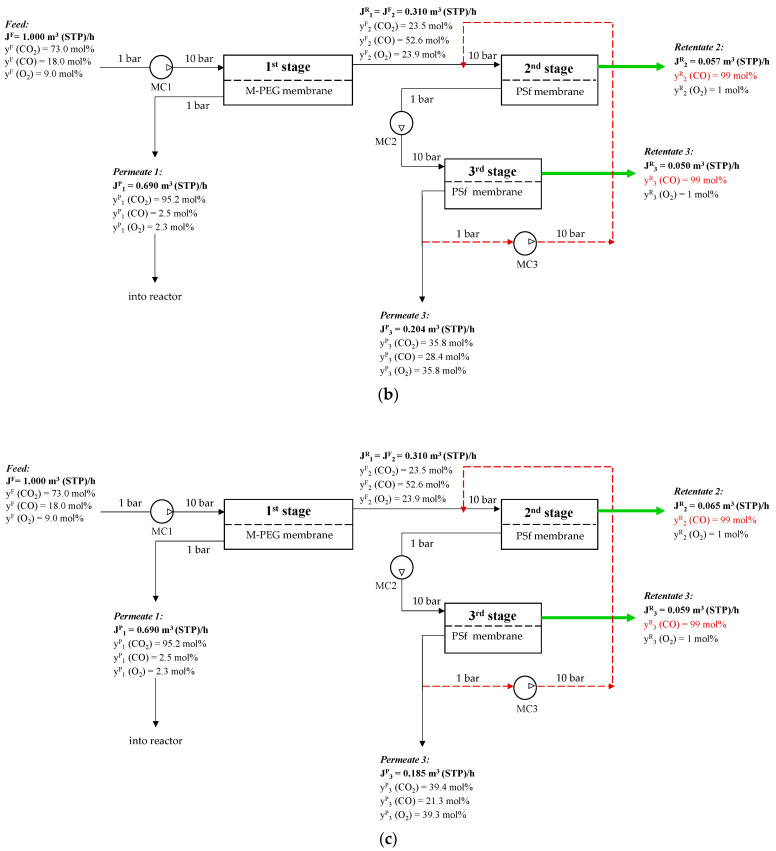
Schematic of CO concentration from a ternary gas mixture of CO_2_/CO/O_2_ after plasma-chemical treatment of CO_2_ by membrane gas separation: (**a**)—20% permeate three-stage way out flow; (**b**)—10% permeate three-stage way out flow; (**c**)—5% permeate three-stage way out flow. (MC1–multistage compressor before first stage, MC2—multistage compressor before third stage, MC3- multistage compressor for recycle).

The proposed three-stage membrane scheme for CO removal with recycling is shown in Figure 10.

To determine the optimal flow chart for this separation process, we need to consider not only the CO recovery rate and the required membrane area, but also the specific energy consumption. The main contributors to the energy consumption in the proposed flow chart are the compression step of the feed stream before the first stage and the permeate streams from the second and third stages. The specific energy consumption for the entire separation process without recycling was 0.13 kW·h per 1 m^3^ of feed. However, as mentioned earlier, this flow chart is not effective in terms of CO recovery. A comparison of the key performance indicators for the recovery and concentration of CO to 99 mol% in the target stream using flow charts with and without recycling is presented in Table 5.

There are a few studies on an integrated approach to separating the CO_2_ splitting product mixture. Most studies focus on adsorption separation and propose models for using different types of zeolites to achieve the target goal. In one study [61], the authors conducted an adsorption separation of CO at variable pressure on zeolites after plasma-chemical treatment of CO_2_ in two stages. In the first stage, FAU-type zeolites were used to extract CO_2_, and in the second stage, BRE-type zeolites separated CO and O_2_. The stream from the first stage contained 98.1% CO_2_ and 1.4% CO, with 0.5% O_2_, which the authors proposed to recycle back to the plasma-chemical reactor. After the membrane separation, a stream with a purity comparable to that of the adsorption method was obtained (Figure 4). Separating the mixture of CO and O_2_ yielded a CO stream with 98.7% purity and a recovery rate of 98%. A similar study was performed in [62], which proposed a scheme involving 10 adsorption columns using different types of zeolites. However, these studies did not take into account the material balance or energy consumption of the proposed system. The PSA process was accompanied by periodic pressure increases/decreases in the columns, which required the continuous operation of the compressors. Additionally, it is known that the slip stream of the product stream in PSA is used to intensify the desorption process. Due to the lack of this data in works [61,62], it is not possible to make a direct comparison between adsorption and membrane separation. According to work [104], the specific energy consumption for concentrating 99% CO using adsorption is 0.27 kWh/m^3^ of feed mixture. Compared to the membrane gas separation system presented in this study, we can conclude that the case with a 20% permeate stream and three-stage flow out is similar to the PSA in terms of specific energy consumption. Further increases in the CO recovery rates increase both the specific energy consumption and required membrane area.

In comparison with previous studies on CO adsorption separation using zeolites, this paper introduces a new approach to CO membrane separation and concentration. The proposed method achieves a 99% CO purity at a total recovery rate of 47.9–69.4%, using only three membrane modules and different types of polymer gas separation membranes (M-PEG and PSF). This allows for the production of a CO stream with 99.0 mol% purity, while using only 0.31–0.83 kWh per 1 m^3^ of feed mixture. It is worth noting that reducing CO concentration in the retentate leads to lower energy consumption and smaller membrane area requirements. For example, oxidative carbonylation of mixtures of CO and O_2_ instead of pure CO is more energy-efficient because it produces a mixture suitable for further catalytic process.

## 5. Conclusions

A conceptual scheme for the separation of CO_2_ plasmolysis products by membrane technology has been proposed. The main objectives are to produce high-purity CO (99 mol%) and recover CO_2_. M-PEG and PSF membranes have been used based on the main tasks. The feasibility and efficiency of using new CO_2_-selective membranes based on oligoethylene glycol methyl ether-substituted polysiloxane (M-PEG) to recover CO_2_ from a ternary gas mixture from the CO_2_ plasmolysis process in the first stage have been discussed. Hollow-fiber gas separation membranes made of polysulfone (PSF) have been selected for the separation of CO and O_2_ at the second stage. For the first time, the permeance of CO through the CO_2_-selective M-PEG membrane and the PSF membrane was measured. It has been shown that the O_2_/CO and CO_2_/CO selectivities are 2.0 and 29.1 for the M-PEG membrane, and 3.04 and 17.2 for the PSF membrane, respectively. Based on the obtained data, a three-stage membrane separation process with recycling has been calculated. The criteria for the first stage are to maximize the CO_2_ concentration in permeate and minimize the loss of CO_2_. Based on mathematical modeling, we have determined that the optimal solution, in terms of energy consumption and membrane area, is to compress the feed stream to 10 atm. According to the calculations, 0.064 m^2^ of M-PEG membrane is required to separate 90% of CO_2_ from the feed stream at a flow rate of 1 m^3^ (STP)/h in the first stage of the scheme. The specific energy consumption for compressing the feed stream to 10 atm was 0.1 kWh per 1 m^3^/h of feed. The first stage permeate stream, which has a concentration of 95.2 mol% CO_2_, is proposed to be recycled back to the CO_2_ plasmolysis reactor. The criteria for the second and third stages of the overall scheme are to maximize CO concentration in the retentate while minimizing CO loss, considering also energy consumption and membrane area. Due to the low CO removal of 21.1% in the three-stage overall process, we proposed a scheme with recycling to increase the CO concentration. An analysis of the correlation between the third stage permeate and the overall CO recovery rate has been conducted. The scheme allows for achieving a CO recovery rate between 47.9 and 69.4%, with specific energy consumption ranging from 30.31 to 0.83 kWh per 1 m^3^ of feed mixture for the separation process. The membrane areas required for M-PEG are 0.1 m^2^, while those for PSF range from 42.5 to 107 m^2^.

## Figures and Tables

**Table 1:** Physical properties of gases [43,59].

Membrane	Membrane Type	Permeability Coefficient, Barrer			Selectivity		Ref.
		CO_2_	CO	O_2_	CO_2_/CO	O_2_/CO	
	Matrimid^®^	8.1	0.5	1.8	16	3.6	[84]
PEI ^1^	Ultem^®^1000B	2.1	0.04	n/a	53	n/a	[85]
PES ^2^	Ultrason^®^E	5.6	0.11	n/a	51	n/a	
PDMS	-	3200 ^a^	500 ^a^	800 ^b^	6.4	1.6	^a^ [79], ^b^ [80]
PTMSP ^3^	-	18200	5400	n/a	3.4		[79]
Pebax 1074	-	87.5	2.9	n/f	30	n/a	[86]

^1^ PEI—polyetherimide; ^2^ PES—polyethersulfone; ^3^ PTMSP—poly(trimethylsilylpropyne).

**Table 2:** Initial data for mathematical modeling.

Parameters	Values
Initial feed gas flow rate, m^3^ (STP)/h	1
Initial feed gas composition, mol%:	
CO_2_	73
CO	18
O_2_	9
Temperature, °C	25

**Table 3:** Gas permeability of PSF and M-PEG membranes for CO_2_, CO, and O_2_, as well as ideal selectivity CO_2_/CO, CO_2_/O_2_, and O_2_/CO.

Membrane	Q_CO2_, GPU	Q_CO_, GPU	Q_O2_, GPU	α_i_ CO_2_/CO	α_i_ CO_2_/O_2_	α_i_ O_2_/CO
M-PEG	867	29.8	60.0	29.1	14.5	2.0
PSF	93.4	5.43	16.5	17.2	5.66	3.04

**Table 4:** Comparison of specific energy consumption and membrane areas under different pressure mode.

Pressure Mode	Up-Membrane Pressure, Atm	Under-Membrane Pressure, Atm	Specific Energy Consumption, kW·h/1 m^3^ Feed	Membrane Area, m^2^ for Feed Flow 1 m^3^/h
I	30	1	0.18	0.02
II	10	1	0.10	0.06
III	1	0.1	0.05	0.64

**Table 5:** Comparison of key parameters for schemes with and without recycling.

Parameters	Without Recycling	With Recycling		
		20/80	10/90	5/95
CO recovery rate, %	21.1	47.9	59.0	69.4
Membrane area, m^2^				
M-PEG	0.064	0.064	0.064	0.064
PSF	12.6	42.5	68.5	107
Specific energy consumption, kW·h per 1 m^3^ of feed				
MC1	0.1	0.1	0.1	0.1
MC2	0.03	0.12	0.21	0.38
MC3	—	0.09	0.18	0.35
Summary, kW·h per 1 m^3^ of feed	0.13	0.31	0.49	0.83

## Data Availability

The original contributions presented in this study are included in the article. Further inquiries can be directed to the corresponding author.
